# Biotechnology strategies with industrial fuel ethanol *Saccharomyces cerevisiae *strains for efficient 1^st ^and 2^nd ^generation bioethanol production from sugarcane

**DOI:** 10.1186/1753-6561-8-S4-O36

**Published:** 2014-10-01

**Authors:** Boris U Stambuk

**Affiliations:** 1 Laboratório de Biologia Molecular e Biotecnologia de Leveduras, Departamento de Bioquímica, Universidade Federal de Santa Catarina, Campus Trindade, Florianópolis SC 88040-970, Brazil

## Background

In Brazil the production of fuel ethanol is based on the fermentation of sucrose from sugarcane by selected industrial *Saccharomyces cerevisiae *yeast strains [1-3], a mature and highly competitive technology. Taking into account that the feedstock costs have a major role in the overall economics of the process, it is expected that more efficient conversions of sucrose into ethanol (1^st ^generation bioethanol) will be of high economical significance. Another promising strategy to improve bioethanol production is the fermentation of the lignocellulosic material present in sugarcane bagasse and leaves (2^nd ^generation bioethanol), a biomass containing large amounts of xylose, a sugar not fermented by *S. cerevisiae *yeast strains [4]. Thus, although the integration of 1^st ^and 2^nd ^generation ethanol production in the Brazilian industry is considered the best option for bioethanol production [5], there are still several drawbacks to fully develop an efficient industrial production system. In this presentation we will show the metabolic and genomic engineering strategies that we are introducing into industrial yeast strains to improve bioethanol production in Brazil.

## Results and conclusion

We have recently show that it is possible to improve sucrose fermentation through genomic and evolutionary engineering strategies that switch the way yeast cells ferment this disaccharide: the active transport and intracellular sucrose hydrolysis allows an increase of 11% in the ethanol yield [6]. We are introducing these modifications into the genome of diploid industrial fuel ethanol yeasts that dominate fermentation processes in Brazil [1,2], showing excellent results. In order to improve the 2^nd ^generation bioethanol production, we have initially screen a panel of several Brazilian industrial *S. cerevisiae *strains for their ability to ferment xylulose (an intermediate in xylose catabolism), and the best xylulose fermenting yeasts were engineered by chromosomal integration of the xylose reductase and xylitol dehydrogenase genes (both from *Scheffersomyces stipitis*), and also the xylulokinase gene from *S. cerevisiae*, under control of strong constitutive promoters. Our results show that the recombinant yeast strains can ferment xylose efficiently, especially under glucose-xylose and sucrose-xylose co-fermentations, highlighting the importance of modifying industrial yeast strains for efficient 1^st ^and 2^nd ^generation bioethanol production from sugarcane.

In an attempt to further improve the industrial recombinant yeast strains, new yeast species isolated from rotten wood in Brazil [7-9] have been also evaluated for the fermentation of xylose and cellobiose (a disaccharide present in cellulose hydrolysates). Cellobiose inhibits the cellulases required for cellulose hydrolysis, and the β-glucosidases used to hydrolyze this disaccharide constitute the highest cost in the enzymatic blend. Our results show that some *Spathaspora *yeast strains are efficient cellobiose fermenters due to active transport of the sugar and intracellular hydrolysis of disaccharide, which has a great potential for lignocellulose fermentation since there will not be glucose present in the hydrolysates to compete with xylose transport by the yeast hexose transporters [4,10]. Some *Spathaspora *yeasts are also efficient xylose fermenting strains due to xylose reductase and xylitol dehydrogenase enzymes with paired co-factor preferences, as well as high-capacity active H^+^-xylose symporters. We have sequenced the genome of the type strain of the xylose-fermenting *S. arborariae *yeast [7], and identify several candidate genes encoding for the enzymes involved in xylose catabolism, as well as genes for sugar transporters. These genes have been already cloned into overexpression plasmids for introduction into *S. cerevisiae *yeasts to further optimize bioethanol production from sugarcane in Brazil.
